# Role of Myo/Nog Cells in Neuroprotection: Evidence from the Light Damaged Retina

**DOI:** 10.1371/journal.pone.0169744

**Published:** 2017-01-18

**Authors:** Alice Brandli, Jacquelyn Gerhart, Christopher K. Sutera, Sivaraman Purushothuman, Mindy George-Weinstein, Jonathan Stone, Arturo Bravo-Nuevo

**Affiliations:** 1 Bosch Institute and Discipline of Physiology, University of Sydney, Sydney, Australia; 2 Philadelphia College of Osteopathic Medicine, Philadelphia, PA, United States of America; 3 Lankenau Institute for Medical Research, Wynnewood, PA, United States of America; Massachusetts Eye & Ear Infirmary, Harvard Medical School, UNITED STATES

## Abstract

**Purpose:**

To identify Myo/Nog cells in the adult retina and test their role in protecting retinal photoreceptors from light damage.

**Methods:**

Light damage was induced by exposing albino rats raised in dim cyclic light to 1000 lux light for 24 hours. In one group of rats, Myo/Nog cells were purified from rat brain tissue by magnetic cell sorting following binding of the G8 monoclonal antibody (mAb). These cells were injected into the vitreous humour of the eye within 2 hours following bright light exposure. Retinal function was assessed using full-field, flash electroretinogram (ERG) before and after treatment. The numbers of Myo/Nog cells, apoptotic photoreceptors, and the expression of glial fibrillary acidic protein (GFAP) in Muller cells were assessed by immunohistochemistry.

**Results:**

Myo/Nog cells were present in the undamaged retina in low numbers. Light induced damage increased their numbers, particularly in the choroid, ganglion cell layer and outer plexiform layer. Intravitreal injection of G8-positive (G8+) cells harvested from brain mitigated all the effects of light damage examined, i.e. loss of retinal function (ERG), death of photoreceptors and the stress-induced expression of GFAP in Muller cells. Some of the transplanted G8+ cells were integrated into the retina from the vitreous.

**Conclusions:**

Myo/Nog cells are a subpopulation of cells that are present in the adult retina. They increase in number in response to light induced stress. Intravitreal injection of Myo/Nog cells was protective to the retina, in part, by reducing retinal stress as measured by the Muller cell response. These results suggest that Myo/Nog cells, or the factors they produce, are neuroprotective and may be therapeutic in neurodegenerative retinal diseases.

## Introduction

Myo/Nog cells belong to a distinct lineage discovered in the blastocyst of the chick embryo [1–5]. They were identified by their expression of mRNA for the skeletal muscle specific transcription factor MyoD, the bone morphogenetic protein (BMP) inhibitor Noggin and the cell surface protein recognized by the G8 monoclonal antibody (mAb)[1, 4–7]. During gastrulation, Myo/Nog cells become widely distributed in small numbers throughout the embryo [1, 3, 8]. Depletion of Myo/Nog cells in the blastocyst results in an inhibition of skeletal muscle differentiation, externalization of organs through the body wall and severe malformations of the central nervous system [1, 3, 8].

Our understanding of Myo/Nog cells was extended when it was discovered that Myo/Nog cells originating in the epiblast are critical for the development of the eye in chick [1, 8]. The first evidence of this role came when Myo/Nog cells tagged within the epiblast of the blastula were detected later in the developing eyecup and lens [1, 8]. Depletion of Myo/Nog cells at this early embryonic period resulted in eye defects such as anophthalmia, microphthalmia, lens dysgenesis and abnormalities in the retina (e.g. retinal folding) [1, 8]. Ocular and other malformations were prevented or reduced in severity with the addition of Noggin or the reintroduction Myo/Nog cells into the embryo, indicating that Myo/Nog cells’ titration of BMP signalling is essential for normal development [1, 3, 8].

Recently, our group described the role of Myo/Nog cells in the developing retina under normal and stressed conditions in neonatal mice [9]. Small numbers of Myo/Nog cells were detected in the neonatal and adult mouse retina.

A model of retinopathy of prematurity (ROP) was used to study the response of Myo/Nog cells to stress[9]. It was discovered that Myo/Nog cells were protective, as depletion of these cells resulted in an increase in photoreceptor death. These studies indicate that Myo/Nog cells have important functions during embryonic and postnatal retinal development.

The aims of the present experiments were to determine whether Myo/Nog cells are present in the retina of the adult rat, examine their behaviour in response to light-induced degeneration of photoreceptors and determine whether increasing their numbers affects retinal function and the Muller cell response to stress.

## Methods

### Animals

Sprague Dawley rats were sourced from the Animal Resource Centre (Perth, WA, Australia). They were raised from birth in controlled scotopic conditions (12 hours at 5–8 lux, 12 hour dark, and 22°C) to 4 to 6 months of age. Normal chow (WEHI, Barastoc, VIC, Australia) and water were available *ad libitum*. All experimental and animal care procedures were approved by the University of Sydney Animal Ethics Committee.

### Treatment groups

There were five treatment groups used to study the effect of Myo/Nog cells (G8 mAb positive cells) on uninjured and light damaged (LD) retinas (control, n = 4; G8+, n = 3, LD, n = 18; LD/PBS, n = 18; LD/G8+, n = 18). Immediately following light induced damage (1000 lux), animals were injected. At the same time point (day 0) non-injured animals were also injected. Seven days after bright-light exposure/injection, the flash ERG measurements were recorded and eyes were harvested for immunohistochemistry. An additional treatment group was used to study the migration of Myo/Nog cells injected in the LD retina (G8+/bisbenzimide, n = 4). Two days following injection of bisbenzimide labeled G8+ cells eyes were harvested for microscopy analysis.

### Exposure to bright light

Rats assigned to light damage groups were exposed to bright continuous light for 24 hours to induce retinal degeneration. Bright light exposure begun at 9:00am, 2 hours after the beginning of the ‘day’ part of the 12h/12h cycle of light in which they were raised. The animals were separated into individual boxes with transparent plexiglass covers. The light source was a cool white fluorescent tube (5000 K, 58W, #8137 Philips, Amsterdam, Netherlands) that delivered 1000 lux measured from the rat’s head height within the box. After a 24-hour exposure to this light, rats were either injected and/or returned to normal housing under the cyclic scotopic (~5 lux) light/dark conditions in which they were raised (above). Seven days after light exposure, the ERG was recorded and the retinas were harvested for examination.

### Preparation for ERG

Rats were dark adapted overnight (12–15 hours) and recordings were performed the following morning. Under dim red light illumination to minimise adaptation, rats were anaesthetised by intraperitoneal injection of ketamine (60 mg/kg) and xylazine (7 mg/kg) (Parnell Manufacturing Pty Ltd, Alexandria, NSW, Australia). Mydriasis was achieved with topical application of atropine sulphate (1.0%) (Bausch & Lomb Australia Pty. Ltd, Macquarie Park, NSW, Australia). Proxymetacaine (0.5%) (Alcon Laboratories Pty Ltd, Frenchs Forest, NSW, Australia) was applied topically for corneal anaesthesia. Corneal hydration was maintained during recordings with Carbomer (2 mg/g) (Novartis Pharmaceuticals, North Ryde, NSW, Australia). A thread was loosely drawn around the eyeball to minimise lid movement. The animal was supported on a platform warmed by internal circulating water at 40°C to maintain body temperature at 37–38°C, as monitored by a rectal temperature probe (Harvard Apparatus, Holliston, MA).

### Full-field ERG recordings

With the animal laid on the warmed platform, the head was positioned with the right eye exposed to a Ganzfeld integrating sphere (Photometric Solutions International, Huntingdale, Victoria, Australia). Once the electrode setup was complete and the dim red light removed, 10 minutes (min) were allowed for stabilisation of conditions before commencement of recording.

The ERG was recorded between a custom-made 4 mm platinum positive electrode lightly touching the cornea and a 2 mm diameter Ag/AgCl pellet electrode (#E206, SDR Clinical Technology, Middle Cove, NSW, Australia) placed in the mouth. Both electrodes were referenced to a stainless steel needle (23 g x 1.25 inch) (Terumo Medical, Somerset, NJ, USA) inserted into the skin of the rump. Signals were recorded with band-pass setting of 0.3–1000 Hz, with a 2 kHz acquisition rate using a PowerLab (4SP system, AD Instruments Pty Ltd, Bella Vista, NSW, Australia).

The light stimuli used to elicit the ERG were brief flashes from light emitting diodes (LEDs). The duration and intensity of the flash were 1–2 ms and -4.4–2.0 log scot cd.s.m^-2^, respectively. Luminous energy was calibrated (IL1700; International Light Research, Peabody, MA) to give rodent (λ_max_ = 502 nm) scotopic (Z-CIE luminosity filter) luminous measures (cd.s.m^-2^).

Eleven intensities of flash were used over the range stated above. At lower flash intensities (-4.4 to -0.3 log scot cd.s.m^-2^), responses were averaged from four flashes delivered at 1Hz. At higher intensities (0.4 to 2.0 log scot cd.s.m^-2^), fewer responses were averaged and inter-stimulus intervals were increased up to 90s. Outcome measures were the amplitudes of the a- and b-waves [10].

### Isolation and injection of Myo/Nog cells

Myo/Nog cells were extracted from the rat brain using the G8 mAb [6]. The brain was rinsed in sterile phosphate buffer saline (PBS), minced in a solution of 0.25% Trypsin-EDTA (Life technologies Australia Pty Ltd, Mulgrave, VIC, Australia) and incubated at 37°C for 15 minutes. An equal volume of DMEM/F-12/HEPES/10% fetal bovine serum (FBS) containing medium (Life technologies Australia Pty Ltd, NSW, Australia) was added to the cell suspension. Following centrifugation, fresh 0.25% Trypsin-EDTA was added to the remaining tissue pieces and the previous step was repeated. Cells that bound IgMs were removed from the cell suspension by incubating with non-specific mouse IgM (375 μg/ml; Abcam Inc., Cambridge, MA) and magnetic beads coated with anti-mouse IgM (Miltenyi Biotec Australia Pty. Ltd, North Ryde, NSW, Australia). Cells that did not bind the magnetic sorting column (those that did not bind either the non-specific IgM or anti-mouse IgM) were resuspended in DMEM/F-12/ HEPES/FBS medium containing the G8 mAb diluted (1:10). Following incubation for 60 min at 37°C and centrifugation, the cells were resuspended in anti-mouse IgM coated magnetic beads diluted 1:5 in PBS. G8+ cells were isolated by magnetic cell sorting and suspended in PBS. The cells were then counted and kept on ice until injection.

Intravitreal injections were performed 2–4 hours following 24 hours of light exposure following a previously published protocol[11]. Rodents were anesthetized with medical grade oxygen mixed with 1.5% isofluorane. The cornea was anesthetised by topical application of 1% proxymetacaine hydrochloride (Allergan, Gordon, NSW, Australia). Gauze soaked in 5% povidone-iodine solution was positioned on the lateral surface of the eye for 30-60s to sterilise the sclera prior to puncturing. A fine needle (30 gauge, Terumo Medical Corporation, Somerset, NJ, USA) was used to puncture the sclera 2 mm posterior to the limbus and tangential to the lateral canthus. A Hamilton needle and syringe (35 gauge, Hamilton syringes, Reno, USA) was used to inject 3 μl of solution. Immediately after injection, a cotton bud was placed on the injection site for 60 seconds and the site was rinsed with sterile 9% saline. Both eyes were injected either with PBS or 6,000 G8+ cells.

All intravitreal injected animals were monitored daily to check for signs of an adverse reaction to the injection. If a cataract or haemorrhage was suspected in the injected eye, the eye was excluded from the study. The proportion of cataract/haemorrhages did not substantial differ between treatment groups (frequency of cataract/haemorrhages: 25% G8+, 20% LD/G8+, 17% LD/Sham).

### Bisbenzimide labeled G8+injection protocol

To determine the final retinal location of injected G8+ cells, live cells were tagged with the nuclei stain bisbenzimide 33342 and injected into the vitreous[12]. G8+ cells were extracted and incubated with filter-sterilized 5 ug/ml bisbenzimide 33342 (Sigma, St Louis, MO, USA) in DMEM/F-12/HEPES/FBS medium for 4 minutes at 37°C and then thoroughly washed in DMEM/F-12/HEPES/FBS medium. Cells were washed and suspended in PBS and kept on ice. 3 uL of G8+/bisbenzimide 33342 labeled cells (6,000 cells) were injected into the eye of LD animals (n = 4). 2 days following the injection, animals were euthanized and eyes were processed for histology.

### Tissue collection

Immediately following completion of the post-treatment ERG, rats were euthanized with an overdose of 60 mg/kg Lethabarb (Virbac, Regent Park, NSW, Australia). A stitch was sown into conjunctiva at the superior aspect of the eye, to provide orientation. Prior to enucleation, the eye was pierced through the anterior chamber with a 25-gauge needle to facilitate fixation.

### Histology

Eyes were fixed by immersion in 4% paraformaldehyde in PBS at 4°C for 2 hours, rinsed in PBS and then cryoprotected overnight in 30% sucrose in PBS. Eyes were embedded in OCT compound (TissueTek, Sakura Finetek Europe, Alphen aan den Rijn, Netherlands) and frozen indirectly in isopentane cooled with liquid nitrogen. Retinas were sectioned at 20 μm, oriented from superior to inferior, using a cryostat (CM1850, Leica, North Ryde, NSW, Australia). Sections passing through the optic nerve head were collected on gelatin and poly-L-lysine coated slides, and stored at -20°C.

Sections were doubled labeled with the terminal deoxynucleotidyl transferase dUTP nick end labeling (TUNEL) technique to detect apoptotic cells (Roche, Basel, Switzerland), and an antibody against glial fibrillary acidic protein (GFAP) (1:1000, Dako, Glostrup, Denmark) to label activated Muller cells. The TUNEL technique used a Tetramethylrhodamine (TMR) labelled dUTP reporter system for 1 hour at room temperature [13]. The G8 mAb (1:50) that binds to Myo/Nog cells, Noggin (1:100, Jackson ImmunoResearch Laboratories Inc., West Grove, PA, USA) and Myosin D (1:20, Jackson ImmunoResearch Laboratories Inc.) antibodies were used to confirm the specificity of the G8 mAB for binding Myo/Nog cells in rodent. Sections were blocked with 10% goat serum (Sigma) in PBS for 30 min at room temperature and incubated with primary antibodies overnight at 4°C in 1% goat serum. Secondary antibodies, including goat anti-rabbit IgG AlexaFluor 488 (1:500; Life technologies, Carlsbad, CA, USA), goat anti-mouse IgG Rhodamine (1:600; Jackson ImmunoResearch Laboratories Inc) or goat anti-mouse IgM rhodamine (1:100; Jackson ImmunoResearch Laboratories Inc), were applied overnight at 4°C. Negative control experiments for the G8 mAb included a non-specific mouse IgM (1:100; Abcam Inc.) or incubation in secondary antibodies only. After washing in PBS, sections were incubated for 2 minutes with the nuclear label bisbenzimide (1:10,000 w/v, Sigma). Sections were mounted in glycerol/gelatin (1:1 v/v, Sigma) and cover-slipped.

Sections were imaged on a Zeiss Axioplan 2 upright microscope (Carl Zeiss, Gottingen, Germany). Images were subsequently adjusted for contrast and brightness using Adobe Photoshop CS4 (Adobe Systems, San Jose, CA). The retinal span was sampled systematically in 400 μm steps along sections cut from superior to inferior. At each field sampled, the thickness of the outer nuclear layer (ONL) was measured and normalised to retinal thickness, measured between the inner limiting membrane (ILM) and outer limiting membrane (OLM). GFAP labeling of Muller cells was quantified by measuring the length of GFAP expression along the Muller cell normalised to the thickness of the retina from the inner to the outer limiting membranes (ILM-OLM). TUNEL+ cells were identified by homogeneous labeling of the nucleus, as described previously [14]. The number of TUNEL+ cells was quantified in the ONL. Counts and measurements were averaged over 3 sections per eye, for 6 animals per group. G8/bisbenzimide cells were identified in unstained sections by using fluorescent microscopy (UV-excitation) and transmitted light to determine the retinal location.

### Statistical analysis

Comparisons between the number of Myo/Nog cells in the LD and control retinas used a two-tailed Student’s t-test. The relationship between Myo/Nog cell counts and TUNEL+ cell counts was analysed using linear regression and Pearson correlation test. Two or more treatment groups were compared using a one-way ANOVA with Dunnett’s post-hoc analysis and a two-way ANOVA with Bonferroni post-hoc testing for a second independent variable. All analysis was performed using statistical software (Graph Pad V5.01, La Jolla, CA, USA).

## Results

### Myo/Nog cells are present in the unstressed retina and their numbers increase following light injury

**Fig 1:**
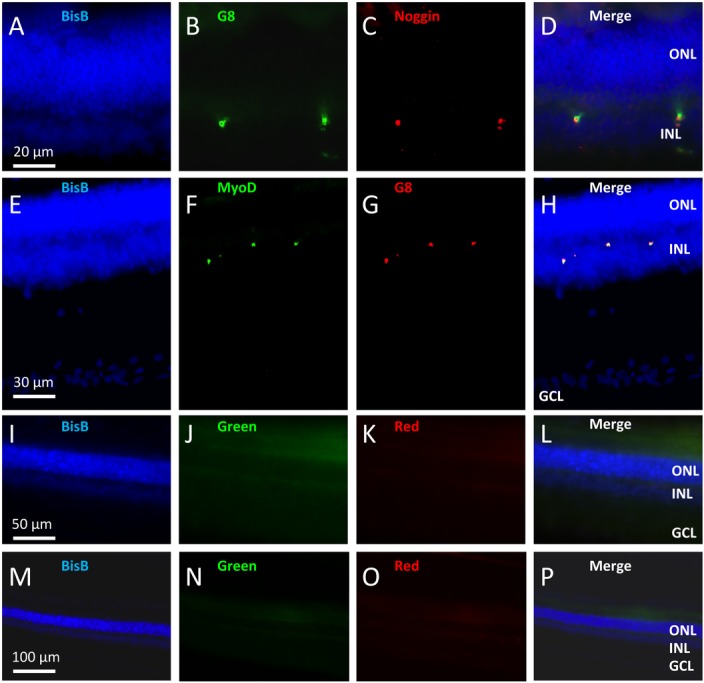
Identification of Myo/Nog cells in the rat retina. Sections of adult rat retinas were double labeled with the G8 mAb and antibodies to Noggin and MyoD to localize Myo/Nog cells. Nuclei were stained with bisbenzimide dye (blue: A, D, E, H, I, L, M and P). Co-labeling with the G8 mAb (green: B and red: G) and antibodies to Noggin (red: C and D) and MyoD (green: F, H) identified Myo/Nog cells in the outer plexiform and inner nuclear layers. Incubation with a non-specific IgM primary antibody and the AlexaFluor 488 IgM secondary antibody showed no visible labeling in the “green channel” (green: J, L) or “red channel” (red: K, L). Incubation with only the AlexFluor 488 IgM secondary antibody showed no visible labeling in either channel (N-P). ONL, outer nuclear layer; OPL, outer plexiform layer; INL, inner nuclear layer; GCL, ganglion cell layer.

**Fig 2:**
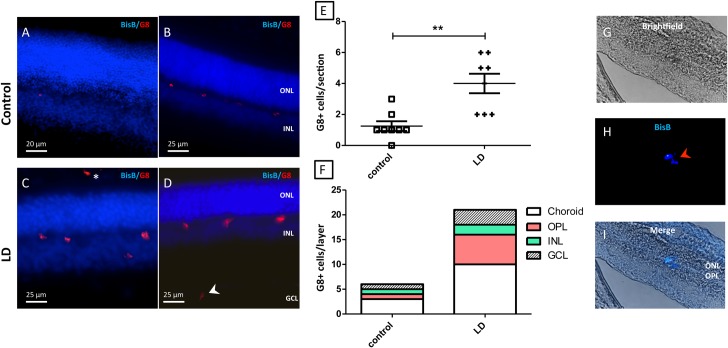
Endogenous Myo/Nog cells increase following light damage. Sections from light damaged and control retinas were labeled with the G8 mAb (red) and bisbenzimide dye (blue). (A, B) Representative section of from a control retina showing the presence of Myo/Nog cells in the OPL. C/D: Representative section of a light damaged retina showing G8+ cells Myo/Nog cells in the OPL, INL, RPE (*) and GCL (˂). E: The numbers of G8+ cells per section of control (squares) and LD retinas More G8+ cells were present in the LD than control retinas. **p = 0.02 (n = 8). F: Distribution of Myo/Nog cells per retinal layer in control and LD retinas. (G-H) LD eyes were injected with Myo/Nog cells that had been prelabeled with bisbenzimide 33342 (blue). At 2 days post-injection, eyes were sectioned and labeled with the G8 mAb. Myo/Nog cells were identified by localizing bisbenzimide. (G) Brightfield image, (H) Fluorescent image showing the presence of a bisbenzimide 33342+/G8+ cell s (red arrow). (I) Merged image showing the location of the bisbenzimide labeled Myo/Nog cell within the OPL. LD, light damage; ONL, outer nuclear layer; OPL, outer plexiform layer; INL, inner nuclear layer; GCL, ganglion cell layer.

**Fig 3:**
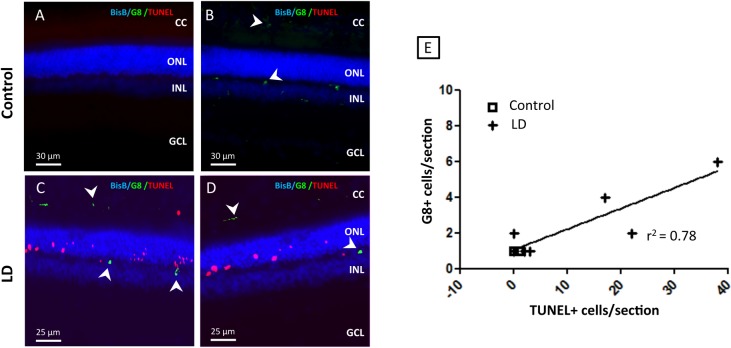
Severity of light-induced cell death correlates with an increase in the number of Myo/Nog cells. Light damage and control retinas were labeled using G8 mAb (green), a marker of Myo/Nog (cells detonated with a white˂), DAPI (blue) a marker of cell nuclei and TUNEL (red) a maker of dying cells. (A,B) Representative image of control retina. Myo/Nog cells (green) are rare, but will occasionally appear in locations like the OPL and CC. We rarely, if ever will observe dying photoreceptors in control animals. (C, D) Representative image of LD retina. In the light damaged retinas, we observed an increase in the TUNEL positive cells (red) and also an increase in the number of Myo/Nog cells in the all layers, but particularly in the OPL and RPE. (E) Correlation between G8+ (Myo/Nog cells) and TUNEL+ cells (dying photoreceptors) in LD retinal section (n = 8). r^2^ = 0.78, p < 0.01 (slope significantly non-zero). CC, choriocapillaris; ILM, inner limiting membrane; INL, inner nuclear layer; ONL, outer nuclear layer; OLM, outer limiting membrane.

Myo/Nog cells in the adult rat retina where detected using the G8 mAb and antibodies to MyoD and Noggin. Consistent with previous studies in chick, mouse, and human [8, 9, 15], the G8 mAb detected cells that also expressed MyoD and Noggin in the rat retina (Fig 1). In control retinas, G8+ cells were found in low numbers in the OPL (Figs 1F–1H, 2B and 3B), INL (Figs 1B–1H, 2A and 2B) and choroid (Fig 3B). Damaging light caused an increased in the numbers of Myo/Nog cells in the choroid (Figs 2C, 3C and 3D) and OPL layers (Figs 2C, 2D, 3C and 3D) and ganglion cell layer (GCL; Fig 2D). There was a significant increase in the number of G8+ cells in the light damaged retina compared to control retinas (Fig 2E). Damaging light also caused an increase in the number of TUNEL+ cells (Fig 3C and 3D) in the outer nuclear layer. This result is consistent with previous studies in which light damage caused the death of photoreceptors [16–18]. G8 and TUNEL labeling did not co-localise and the numbers of the two cell classes positively correlated (Fig 3E), indicating that Myo/Nog cells increased in number as the photoreceptors die.

Extracted G8+ cells were incubated with bisbenzimide 33342, a cell tracking dye and injected in light damaged eyes. Some G8+ cells labeled with bisbenzimide dye prior to injection were detected in the retina. In Fig 2G–2I we observe one of these cells, a G8+/bisbenzimide 33342 cell, within the OPL (Fig 2G–2I). This is a similar region to were endogenously located G8+ cells in light damaged retinas (Figs 2C, 2D, 3C and 3D) are usually found. This finding indicates that G8+ cells are capable of migrating from the vitreous into an injured retina.

### Myo/Nog cells mitigate functional loss following light injury

**Fig 4:**
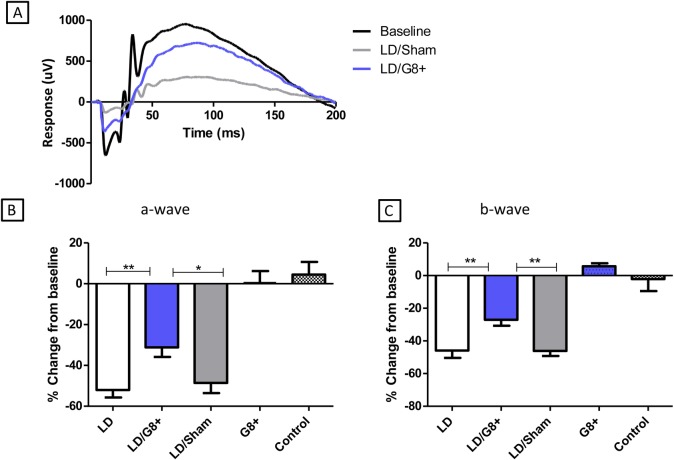
Intravitreal injection of Myo/Nog cells partially preserved visual function after light damage. Visual function was assessed using the ERG 7 days after light damage. The a-wave and b-wave amplitudes in response to bright flash were normalised to baseline (before light damage) bright flash responses and averaged for each treatment group. Control animals suffered no injection and no light damage. G8+ animals suffered no light damage but were injected with purified G8+ cells and assessed 7 days after injection. LD stands for Light damage. (A) Representative ERG traces from eyes with LD alone, LD eyes injected with PBS (LD/sham) and LD eyes injected with G8+ (LD/G8+). (B) Mean change in a-wave amplitudes for retinas from the 5 experimental groups, from before treatment. Error bars show SEMs. Control rats that did not receive light damage showed no significant change in the a-wave between the two ERG measurements (n = 4). Control rats that did not receive light damage, but were injected with G8+ cells also showed no significant change in the a-wave between the two ERG measurements (n = 3). Visual function, as measured by the a-wave, was significantly improved following light damage with injection of G8+ cells (n = 18) compared to LD alone (n = 18) and LD/sham (n = 18) (LD/G8+: vs. LD ** p < 0.01, vs. LD/Sham * p < 0.05).

Light injury severely reduced the a-wave and b-wave components of the ERG measured 7 days following return to scotopic light (Fig 4). These effects are quantified for the a-wave in Fig 4B. Light damage induced approximately a 50% reduction in the amplitude of the a-wave. Intravitreal injection of brain-derived G8+ cells 7 days following light damage improved the ERG a-wave to only a 20% loss of function. A sham intravitreal injection did not improve the a-wave.

Mean b-wave amplitudes for retinas from the 5 experimental groups, normalised to the mean b-wave amplitude before treatment. Error bars show SEMs. Control rats that did not receive light damage or the ones that did not received light damage, but were injected with G8+ cells, showed no significant change in the a-wave between the two ERG measurements. Visual function, as measured by the b-wave, was significantly improved with injection of G8+ cells compared to LD alone and LD/sham (LD/G8+ vs. LD** p < 0.01, vs. LD/Sham* *p < 0.01.

The same analysis for the b-wave showed the same trends (Fig 4C). These ERG analyses demonstrate that intravitreal addition of Myo/Nog cells improves retinal function.

The injection of Myo/Nog cells in normal eyes showed no aberrations in retinal function when tested at 7 days as demonstrated in the a-wave (Fig 4B) or b-wave (Fig 4C). Suggesting that Myo/Nog cells are harmless to the retina under homeostatic conditions and beneficial when the retina is injured.

### Effects of intravitreal injection of Myo/Nog cells on photoreceptor survival

**Fig 5:**
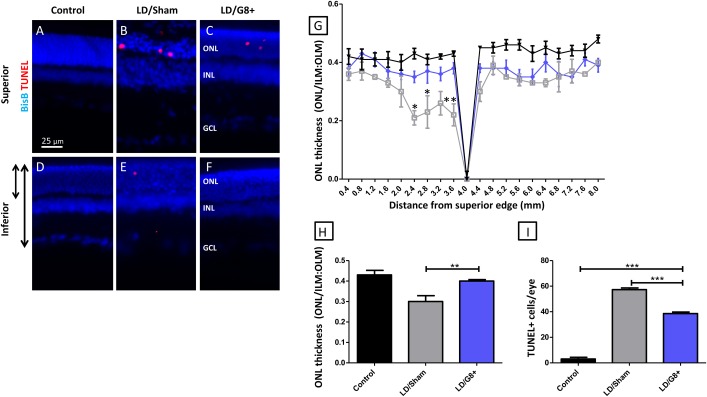
Myo/Nog cell injections mitigated photoreceptor loss. (A-F) Representative sections from superior and inferior retina of non-light damaged control (control), light damage + injection of PBS (LD/Sham) and light damage + injection of g8+ cells (LD/G8+). Dying cells were labeled with TUNEL (red) and cell nuclei were stained with bisbenzimide dye (blue). The arrows at the left of D show how the thickness of the ONL (shorter arrow) and of the nuclear layers of the retina (longer arrow) was measured. Retinas without light damage showed no labeled cells with TUNEL reagents (A and D). TUNEL+ cells were mostly present in the ONL of retinas with light damage in the superior retina when injected with PBS or G8+ cells (B, C and E). TUNEL+ cells were present in the sham injected eye (E) but not present in these control and G8+ injected sections (D and F). The ONL was disorganized in the LD/Sham retina, but not in the control and LD/G8+ retinas. (G) Normalized ONL thickness for the non-light damaged) control (black line), LD/Sham (Gray line) and LD/G8+ (blue line) groups from superior to inferior areas of the retina. Error bars show SEMs. Significant difference in retinal thickness were seen between LD/Sham and LD/G8+; * p < 0.05; **p<0.01. (H) Normalised ONL thickness for control, LD/Sham and LD/G8+ groups. Error bars show SEMs. The ONL is significantly thicker in LD retinas treated with G8+ cells than those injected with PBS. No difference was seen in ONL thickness between control and LD/G8+ sections. **p<0.01. (I) Number of TUNEL+ cells in the ONL/section for the same three groups. Error bars show SEMs. Significantly more TUNEL+ cells were present in the LD retinas than control retina (asterisk p value). Fewer TUNEL+ cells were present in the retinas injected with G8+ cells than those injected with PBS (asterisk p value). (G-I: Control = 5, LD/Sham = 4, LD/G8+ = 5). ***p < 0.001; ** p < 0.01. ILM, inner limiting membrane; INL, inner nuclear layer; ONL, outer nuclear layer; OLM, outer limiting membrane.

The effects of Myo/Nog cells on photoreceptor survival were assessed morphologically and by the number of dying photoreceptor cells. The thickness of the ONL was measured to assess the number of photoreceptors remaining following light damage. The ONL was thinner 7 days following light damage (Fig 5B and 5E). Consistent with our previous studies the thinning was markedly worse in the superior retina (Fig 5B and 5C)[17, 18]. Intravitreal injection of G8+ cells mitigated this thinning (Fig 5C and 5F). Fig 5G and 5H show the trend in ONL thickness (photoreceptor survival) quantitatively. In the control retina, ONL thickness normalised to retinal thickness was ~0.4, confirming previous reports[18] (Fig 5H). Within the superior retina, where light damage effects on photoreceptor survival is greater than in the inferior retina, there was statistical difference between ONL thickness of sham injected and Myo/Nog cell injected light damaged retinas at 2.0,2.4 and 3.6 mm from the superior edge (Fig 5G). Light damage reduced the overall ONL thickness to 0.3 and intravitreal injection of G8+ cells mitigated this reduction (Fig 5H).

TUNEL+ (dying) cells were rare in the control retina and increased in the light-damaged retina where they localized mostly in the ONL (Fig 5B, 5C and 5E). Fig 5I shows the trends in TUNEL labeling (photoreceptor death) quantitatively, with the LD/Sham group containing the highest amount of TUNEL cells (Fig 5B and 5E). Intravitreal injection of G8+ cells mitigated this increase and these eyes contained ~30% fewer TUNEL+ cells than light damaged sham injected eyes (Fig 5C, 5F and 5I).

### Effects of light damage and intravitreal injection of G8+ cells on retinal stress

**Fig 6:**
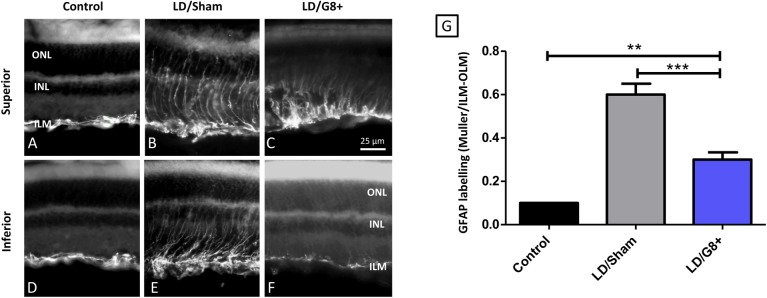
Myo/Nog cell injections mitigated retinal stress following LD, as measured by GFAP expression. (A-F) Representative sections from superior and inferior retina of control (non-light damaged) and light damaged eyes injected with PBS (LD/Sham) or G8+ cells (LD/G8+). Sections were labeled with an antibody to GFAP (white). ILM–inner limiting membrane; INL- inner nuclear layer; ONL–outer nuclear layer; OLM–outer limiting membrane. (G) Normalised length of GFAP labeling for control as measured by the length of the labeled Muller cells normalized to the length of the nuclear retina. Error bars show SEMs. Non-LD retinas had significantly shorter GFAP+ labeled Muller cells than LD retinas (asterisk with p value). Injection of G8+ cells following LD had significantly shorter Muller cells than LD retinas injected with PBS. (Control, n = 5; LD/Sham, n = 4; LD/G8+ = 9). *** indicates p < 0.001; ** indicates p < 0.01.

The intrinsic neuroglia of the retina, the Muller cells, respond to various forms of injury, including bright light exposure, by increasing their expression of GFAP[19]. In the unstressed rat retina, Muller cells do not express GFAP and expression of GFAP is limited to astrocytes at the inner surface of the retina (Fig 6A and 6D). Light damage has been shown to cause an elevation in GFAP expression in Muller cell processes and at its most extreme, GFAP expression extends from the inner to outer limiting membrane (Fig 6B and 6E). The length of GFAP expression along the radial process can be quantified and has been shown to correlate to level of retinal damage[20].

The length of Muller cells’ staining by GFAP was quantified in each experimental animal and normalized to the thickness of the retina measured from the ILM to the OLM. The labeling on the Muller cells was minimal in control retinas and was increased by light damage, extending ~60% of the retinal thickness (Fig 6G) and intravitreal injection of G8+cells mitigated the increase in GFAP expression, to ~25% of retinal thickness.

## Discussion

The present experiments elucidate several features of Myo/Nog cells and their relationship to retinal stability. First, Myo/Nog cells, identified by expression of the G8 antigen, MyoD and Noggin, are present in low numbers in the unstressed, adult rat retina. This study extends our previous reports documenting their presence in the retinas of the chick embryo and neonatal and adult mouse [1, 8, 9]. Second, the number of Myo/Nog cells in the retina increased when the photoreceptors were damaged by exposure to damaging levels of light. Third, Myo/Nog cells were found near the layer of dying photoreceptors in the OPL or RPE layers. Fourth, intravitreal injection of Myo/Nog cells harvested from the brain mitigated the loss of function and death of photoreceptors induced by light damage. And finally, Myo/Nog cells reduced the stress associated with light damage, as evidenced by a reduced Muller cell response.

Exposure to damaging light causes a degeneration of the retina that is specific to photoreceptors [21]. This form of retinopathy also induced an increase in Myo/Nog cells, which correlated with the loss of photoreceptors and the thinning of the outer nuclear layer. Their accumulation near the site of cell death in the rat retina is consistent with the attraction of Myo/Nog cells to sites of injury in the neonatal mouse retina [9]. Myo/Nog cells have also been observed to concentrate at sites of injury in the embryo, skin, lens and tumors [15, 22, 23]. Additional analyses are needed to establish whether the increase in Myo/Nog cells in the stressed retina is due to the proliferation of resident Myo/Nog cells or their migration into the retina from neighboring tissues or the vasculature.

Prolonged exposure to bright light is a reliable, predictable and well-characterized model of retinopathy that affects photoreceptors[24, 25]. This model was used to test whether exogenous Myo/Nog cells harvested from the brain and injected into the vitreous, affect tissue damage and function. Addition of Myo/Nog cells reduced cell death and consequently, improved retinal function as measured by ERG analyses. In parallel with these neuroprotective effects, intravitreal injection of Myo/Nog cells mitigated retinal stress that was revealed by a decrease in the extent of GFAP staining, cell death and reducing the stress response of the Muller cells.

Mitigation of retinal stress suggests that Myo/Nog cells act; either directly or indirectly, on Muller cells that have been shown to release protective factors to promote photoreceptor survival following light induced degeneration[26–28]. Thus, the neuroprotective function of Myo/Nog cells may be secondary to their effect on Muller cells; however, additional studies are required to rule out a direct influence on photoreceptors.

Some Myo/Nog cells that were injected into the vitreous were found within the retina, and therefore, had penetrated the inner limiting membrane (ILM). This was unexpected, as other studies in adult rodents have shown that mature cells injected into the vitreous are typically prevented from migrating into the retina, due to the presence of the ILM [29–31]. However in light induced damage, the blood retinal barrier becomes permeable and there is evidence the inner retina can also be damaged hence making it possible for exogenous cells to enter the retina [32–34]. Regardless of whether Myo/Nog cells influence Muller cells or photoreceptors externally, or within the retina itself, it is likely that the effect involves release of factors that diffuse locally within the tissue.

A potential mediator of Myo/Nog cells’ neuroprotective effect is Noggin, a BMP inhibitor[8]. Noggin is released from Myo/Nog cells during development and in adult tissues, including eyes of humans and mice [1, 3, 8, 9, 15, 22]. This release is critical for morphogenesis, eye development and skeletal muscle differentiation [1, 8]. Overexpression of Noggin has been shown to act as a neuroprotectant in rodent models of stroke[35–37]. Astrocytes, Muller cells and microglia exhibit a gliotic reaction in response to BMPs, and Noggin circumvents gliosis [36–40]. Thus, Myo/Nog cells’ release of Noggin may be at least partially responsible for the reduction of Muller cell stress response.

Previous studies have shown that Myo/Nog cells in the lens proliferate, migrate to sites of injury, synthesize skeletal muscle proteins and display a myofibroblast phenotype [15, 23]. Additionally, myofibroblasts are present in contractile membranes that can detach the retina in diabetic retinopathy or proliferative vitreoretinopathy[41, 42]. Although contractile membranes were not evident in the present study, the ability of Myo/Nog cells to develop into myofibroblasts must be considered when gauging their therapeutic potential for neuroprotection in the retina. Therefore, identification of the factors released by Myo/Nog cells that mediate neuroprotection may be a more viable approach for the treatment of retinopathy.

In conclusion, this study documents the protective action of intravitreally injected Myo/Nog cells for photoreceptors damaged by light. Identification of the neuroprotective factor(s) released by Myo/Nog cells may lead to new therapeutic approaches to preserve photoreceptors and dampen gliosis.
